# Vitamin D and colorectal cancer: Chemopreventive perspectives through the gut microbiota and the immune system

**DOI:** 10.1002/biof.1786

**Published:** 2021-09-24

**Authors:** Emanuele Rinninella, Maria Cristina Mele, Pauline Raoul, Marco Cintoni, Antonio Gasbarrini

**Affiliations:** ^1^ UOC di Nutrizione Clinica, Dipartimento di Scienze Mediche e Chirurgiche Fondazione Policlinico Universitario A. Gemelli IRCCS Rome Italy; ^2^ Dipartimento di Medicina e Chirurgia Traslazionale Università Cattolica Del Sacro Cuore Rome Italy; ^3^ UOSD di Nutrizione Avanzata in Oncologia, Dipartimento di Scienze Mediche e Chirurgiche Fondazione Policlinico Universitario A. Gemelli IRCCS Rome Italy; ^4^ Scuola di Specializzazione in Scienza dell'Alimentazione Università di Roma Tor Vergata Rome Italy; ^5^ UOC di Medicina Interna e Gastroenterologia, Dipartimento di Scienze Mediche e Chirurgiche Fondazione Policlinico Universitario A. Gemelli IRCCS Rome Italy

**Keywords:** colorectal cancer, gut microbiota, immune system, inflammation, vitamin D

## Abstract

Vitamin D and its receptor are involved in health and diseases through multiple mechanisms including the immune system and gut microbiota modulations. Gut microbiota variations have huge implications in intestinal and extra‐intestinal disorders such as colorectal cancer (CRC). This review highlights the preventive role of vitamin D in colorectal tumorigenesis through the effects on the immune system and gut microbiota modulation. The different associations between vitamin D, gut microbial homeostasis, immune system, and CRC, are dissected. Vitamin D is supposed to exert several chemopreventive effects on CRC including direct antineoplastic mechanisms, the effects on the immune system, and gut microbiota modulation. Large clinical studies with a randomized design, are required to confirm the role of vitamin D in CRC, confirming its key role in the complex interplay between the gut immune system and microbiota.

**Abbreviations**
1,25‐D: 1,25‐dihydroxyvitamin D
AMP: antimicrobial peptides
CIMP: CpG island methylator phenotype
CRC: colorectal cancer
CST5: cystatin D gene
EGFR: epidermal growth factor receptor
HDAC4: histone deacetylase 4
IBD: inflammatory bowel disease
IFN: interferon
IGF: insulin growth factor
IL: interleukin
ILC3: type 3 innate lymphoid
KO: knock‐out
MSI: microsatellite instability
MYCBP: myc binding protein
NF‐κB: nuclear factor kappa‐light‐chain‐enhancer of activated B cells
PAMP: pathogen‐associated molecular patterns
PUMA: p53‐upregulated modulators of apoptosis
ROS: reactive oxygen species
SCFA: short‐chain fatty acid
spp: species
Treg: T regulatory
UV: ultraviolet
VDR: vitamin D receptor

## 1 INTRODUCTION

Vitamin D is a liposoluble vitamin available in two forms, D2 (ergocalciferol) and D3 (cholecalciferol). In humans, it is biosynthesized from a derivative of the cholesterol in the skin under the influence of solar ultraviolet (UV)‐B radiation.^1^, ^2^ Vitamin D3 is also present in dietary sources including animal foods such as fatty fish, liver, milk, eggs, and dietary supplements. Both forms of vitamin D are converted to 25‐hydroxyvitamin D in the liver; then, 25‐hydroxyvitamin D passes through the blood to the kidneys, where it is further modified to 1,25‐dihydroxyvitamin D (1,25‐D), or calcitriol, the active form of vitamin D in the body.^3^ As reported in research studies, vitamin D status is assessed by different variables such as serum vitamin D, tissue vitamin D, and vitamin D gene receptor (VDR).^4^ Serum 25‐hydroxyvitamin D level is widely recognized as a biomarker in determining the effect of vitamin D status in the clinical setting.^5^, ^6^ Vitamin D is first known for its traditional role in bone metabolism, but it has other important biological functions including modulation of the immune system and anti‐carcinogenic effects.^7^ Recent meta‐analyses of case–control studies, nested case–control studies, and cohort studies assessed the association between serum vitamin D and the risk of various types of cancer.^8^, ^9^, ^10^ Moreover, vitamin D and its VDR are known to participate in gut microbiota modulations in health and diseases.^11^ The gut microbiota is an extremely rich and diverse ecosystem inside the gastrointestinal tract, containing more than 10^14^ microorganisms.^12^ Gut microbiota variations have huge implications in intestinal and extra‐intestinal disorders such as metabolic diseases and colorectal cancer (CRC).^13^ In this context, this review aims to highlight the role of vitamin D in colorectal tumorigenesis through gut microbiota modulation and the effects on the immune system. We dissect the different associations between vitamin D, gut microbial homeostasis, immune system, and CRC, to depict the potential chemopreventive role of vitamin D in CRC.^13^, ^14^

## 2 GUT MICROBIOTA, IMMUNE SYSTEM, AND CRC

The human microbiota is a complex ecosystem inhabiting the human body and harboring more than 100 trillion microorganisms, most of which colonize the gastrointestinal tract, and specifically the colon, where bacterial density ranges between 10^11^ and 10^12^ cells per milliliter. Gut microbiota shows several and yet poor‐known functions such as nutrient metabolism, maintenance of the gut mucosal barrier, and immunomodulation. Alterations of this ecosystem are nowadays recognized in several diseases, among which cancer, and in particular colon cancer.^13^, ^14^ Compositional gut bacteria variations between fecal samples of CRC patients and healthy volunteers have been shown in numerous studies.^15^ A recent meta‐analysis of fecal metagenomes reveals global microbial signatures that are specific for CRC.^16^ In particular, in CRC patients, the abundance of butyrate producers decreases while opportunistic pathogens increase. This constitutes a major structural imbalance of gut microbiota. Butyrate is a short‐chain fatty acid (SCFA) produced by the bacterial fermentation of dietary oligosaccharides and it has a key role in the gut homeostasis; it stimulates mature colonocytes and inhibits malignant cells; moreover, it lowers luminal pH and maintains a low O2‐tension, favorable to anaerobic commensal bacteria, reducing the risk of an expansion of the pathogen Enterobacteriaceae. In the lamina propria, butyrate induces differentiation of Foxp3+ T regulatory (reg) cells and the expression of interleukin (IL)‐10, an anti‐inflammatory cytokine maintaining immune tolerance and immune‐homeostasis.^17^, ^18^ Thus, butyrate‐producing bacteria contribute to gut barrier function, reducing gut inflammation.^19^ In turn, a reduction in butyrate‐producers may induce an imbalance of gut microbiota, endotoxin, and bacterial products translocation, activating an immune response in the colonic mucosa.^20^ More in‐depth, the gut microbiota of the CRC patients is characterized by enrichment in *Bacteroides fragilis*, *Enterococcus*, *Escherichia/ Shigella*, *Klebsiella*, *Streptococcus*, and *Peptostreptococcus* and a decrease in *Roseburia* and other butyrate‐producing bacteria of the family *Lachnospiraceae*, compared with healthy subjects.^15^ On the other hand, the gut microbiota of the healthy subjects has higher levels of *Bacteroides vulgatus* and *Bacteroides uniformis* compared with CRC subjects. Also, Shen et al.^21^ demonstrated a higher abundance of Proteobacteria and a lower abundance of Bacteroidetes, in colorectal adenomas cases compared to the normal colonic mucosa, suggesting a peculiar inflammatory microbial milieu in CRC precursors. Interestingly, the gut microbiota of CRC patients is enriched in *Fusobacterium* spp, and in particular *Fusobacterium nucleatum*, a periodontal pathogen, whose role in CRC has been extensively debated.^14^, ^22^ In detail, preclinical studies reported a heavy enrichment of CRC tissues in FN, which is associated with genetic and epigenetic alterations such as CpG island methylator phenotype (CIMP) status, microsatellite instability (MSI), and mutations in BRAF, KRAS, TP53, TGF‐β, CHD7, and CHD8.^23^, ^24^ From a molecular point of view, *F. nucleatum* may express its oncogenic potential through the adhesin FadA, activating the E‐cadherin/β‐catenin and Wnt (7a, 7b, 9a) oncogenic pathways, and promoting the synthesis of pro‐inflammatory cytokines such as IL‐6, IL‐8, and IL‐18 as well as TNF‐α and reactive oxygen species (ROS) with subsequently DNA damage.^25^, ^26^ Also, *F. nucleatum* colonizes the tumor environment through the lectin Fap2, binding the host polysaccharide Gal‐GalNAc, which is overexpressed in colorectal adenocarcinoma and metastases.^27^ Fap2 mediates the impairment of host‐antitumor immunity binding and activating TIGIT, an immunoregulatory signaling receptor suppressing T cells and NK cells activation, thus reducing the control of CRC cells by the immune system.^28^, ^29^ Fap2 also induces tumor proliferation through lymphocyte apoptosis.^30^ All the overmentioned findings reveal that alterations in CRC microbiota may contribute to CRC development. Wong et al. went further by giving germ‐free C57BL/6 mice with stool from five patients with CRC or five controls. They collected intestinal tissues and performed histology analysis and 16S ribosomal RNA gene sequencing analysis of feces from mice, showing that gavages of fecal samples from patients with CRC promoted intestinal carcinogenesis.^31^ According to several animal studies, antibiotics eliminate carcinogenic bacteria in mice models.^32^, ^33^ Hence, antibiotic administration could theoretically protect against tumor proliferation. Nevertheless, in humans, antibiotic use is closely linked with an increased risk of CRC incidence.^34^, ^35^ We can hypothesize that in more complex ecosystems, antibiotic treatment exacerbates dysbiosis, by reducing gut microbiota diversity and richness of specific species leading to an increased risk of pathogenic bacterial colonization.^36^

## 3 VITAMIN D, IMMUNE SYSTEM, AND THE GUT MICROBIOTA

Given the long‐term exposure of the colonic mucosa to external agents and the gut microbiota, the elevated load in pathogen‐associated molecular patterns (PAMPs) continuously elicits the immune response. The gut (and systemic) immune homeostasis is thus preserved by regulatory factors such as microbial richness, anti‐inflammatory cytokines, and immune regulatory cells.^37^ In this sense, 1,25‐D, binding to its receptor, could represent a regulatory biofactor, affecting the immune system both directly and through gut microbiota modulation. VDR belongs to a superfamily of nuclear receptors that transduce hormonal signals from the immediate environment and participate in the activation of several genes.^38^ The VDR is expressed in at least 30 different target tissues including bone, kidney, blood, breast, prostate, gut, activated B‐ and T‐ lymphocytes, monocytes, and keratinocytes; this widespread expression may largely explain the pleiotropic effects of vitamin D.^38^ In the colonic mucosa, it has been assessed that VDR stabilizes cell tight junctions between the intestinal epithelial cells inducing the expression of cell junction proteins such as ZO‐1, E‐cadherin, and occludin.^39^, ^40^ Moreover, nuclear VDR is directly involved in the regulation of nuclear factor kappa‐light‐chain‐enhancer of activated B cells (NF‐κB) activation, a pathway essential for inflammatory response^41^; indeed, VDR absence leads to reduced levels of I‐kappa‐B‐alpha protein, the endogenous inhibitor of NF‐κB.^42^ Vitamin D has a key role in both the innate and the adaptive immune systems.^43^ 1,25‐D can stimulate the expression of β‐defensins and cathelicidins,^44^, ^45^ the main classes of antimicrobial peptides (AMPs)^46^—key components of innate host defense^47^—by interacting with VDR. In addition, 1,25‐D enhances immune homeostasis decreasing Th1/Th17 CD4+ T cells activity, enhancing Treg activity, downregulating T cell‐driven IgG production, and inhibiting dendritic cell differentiation^48^ in the intestinal lamina propria. VDR gene acts as a key host modulator of the gut microbiome, even if it is not expressed by prokaryotes cells.^49^ Hence, the effects of vitamin D and its receptor on gut microbiota are considered as indirect through the host immune system.^50^ A recent genome‐wide host–microbiota association study^51^ identified the VDR gene among the most significant loci that were associated with overall gut microbial variation and abundance of individual bacteria, as well as functions of gastrointestinal and immune‐related tissues and cells. In VDR knock‐out (KO) (Vdr−/−) mice, feces are depleted in *Lactobacillus* and enriched in *Clostridium* and *Bacteroides*; *Alistipes* and *Odoribacter* abundances are significantly decreased whereas *Eggerthella* quantity is increased. Moreover, the intestinal‐specific deletion of VDR could be associated with a decrease in butyrate‐producing bacteria. In mice, vitamin D deficiency (combined with a high‐fat diet) may cause dysbiosis, increased gut permeability, mucosal collapse, and systemic inflammation leading to insulin resistance and hepatic steatosis.^52^ In humans, a recent randomized controlled trial enrolling 26 healthy overweight adults evaluated the effects of vitamin D supplementation on the fecal microbiota of two groups (treatment vs. placebo).^53^ The vitamin D group had a higher abundance of genus *Lachnospira*, and a lower abundance of genus *Blautia* than the placebo group. Moreover, individuals with 25‐hydroxyvitamin D > 75 nmol/L had a higher abundance of genus *Coprococcus* and lower abundance of genus *Ruminococcus* compared to those with 25‐hydroxyvitamin D < 50 nmol/L, suggesting potential beneficial effects of vitamin D supplementation.^53^ A recent study of 80 otherwise healthy vitamin D‐deficient women^54^ measured serum 25‐hydroxyvitamin D levels in the blood before and after vitamin D supplementation. Vitamin D supplementation significantly increased gut microbial diversity. Specifically, the Bacteroidetes to Firmicutes ratio increased. At the genus level, significant variations in *Bacteroides* and *Prevotella*, along with the abundance of the health‐promoting probiotic taxa *Akkermansia* and *Bifidobacterium*, indicated a beneficial variation in enterotypes following supplementation. Interestingly, comparing samples, researchers found more pronounced changes in abundance of major phyla in those of responders. A recent cross‐sectional study conducted in 567 North Americans found that men with higher levels of 1,25‐D had greater α‐diversity (the diversity of microbial species within an individual) and β‐diversity (the difference in microbial composition between two samples) than counterparts with lower levels, even after adjusting for other covariates of microbial diversity such as age, geographical origin, race, PPI, and antibiotic use.^55^ Another systematic review of animal and human studies confirmed a significant association between vitamin D status and β‐diversity.^56^ The beneficial role of vitamin D on gut microbial modulation is also confirmed by the evidence that men with elevated 1,25‐D plasmatic levels are more likely to possess butyrate‐producing bacteria.^55^

## 4 CHEMOPREVENTIVE EFFECTS OF VITAMIN D AND THE COMPLEX NETWORK OF GUT MICROBIOTA AND IMMUNE SYSTEM

**FIGURE 1:**
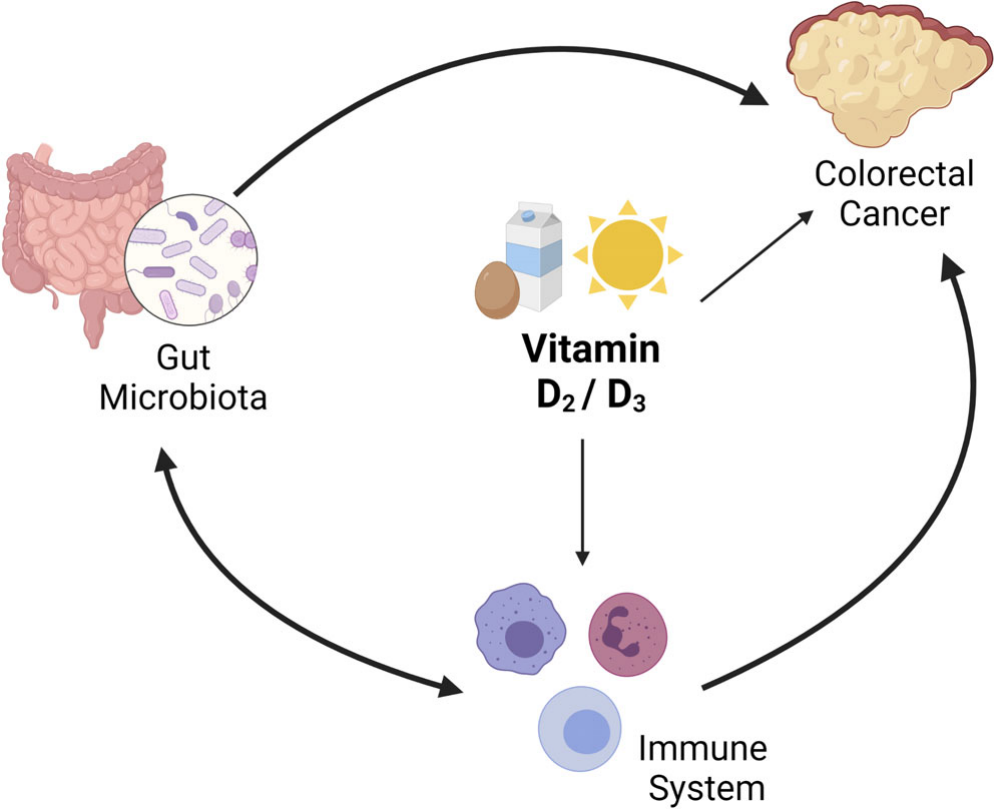
The chemopreventive effects of vitamin D: vitamin D could affect CRC both directly and through the immune system. Gut microbiota and immune system are also interconnected, both of them influencing CRC development and progression

Vitamin D is supposed to exert several chemopreventive effects on CRC (Figure 1).

### 4.1 Direct antineoplastic effects

Vitamin D provides a direct preventive action in CRC onset and progression: its binding to VDR induces several intracellular and intranuclear pathways linked to growth, differentiation, and apoptosis. Transcriptomic studies in CRC cell lines identified hundreds of genes involved in DNA synthesis, apoptosis, and intracellular signals activated by VDR.^57^, ^58^ In detail, the antiproliferative effects of Vitamin D in CRC includes the VDR binding to β‐catenin (preventing the activation of TCF7L2 complexes in the nucleus), the upregulation of the protein CDH1 (E‐cadherin), a transmembrane protein whose intracellular tail attracts β‐catenin, and the inhibition of the proto‐oncogene MYC (upregulated by the WNT/β‐catenin pathway). Moreover, 1,25‐D regulates and inhibits the activity of the epidermal growth factor receptor (EGFR) gene and the signaling activity of insulin growth factor (IGF) 2 protein.^58^, ^59^ Vitamin D has also a regulatory epigenetic action, controlling the histone demethylases KDM6B and activating the cystatin D (CST5) gene. Cystatin D, in turn, inhibits several cysteine proteases of the cathepsin family, reducing migration and growth in CRC cells.^60^ Another indirect activity of 1,25‐D is the regulation of miR‐22 ‐a short RNA molecule targeting histone deacetylase 4 (HDAC4) and myc binding protein (MYCBP)‐ enhancing its antiproliferative and anti‐migratory effect.^61^ Vitamin D activity could also mediate the potential chemopreventive effect of estrogen replacement therapy in postmenopausal women. VDR is a known estrogen‐responsive gene: estradiol administration results in significant changes of estrogen‐responsive genes and seems to increase VDR signaling in the colorectal epithelium of postmenopausal women.^62^ A recent study confirmed that vitamin D intake could have a clinical impact in advanced or metastatic CRC patients undergoing chemotherapy, in a median follow‐up of 2 years.^18^ During the study, half of the patients were given a daily dose of 400 IU of vitamin D. The other group first took two 4000 IU capsules per day for 2 weeks, then one 4000 IU capsule daily. In both groups, the course of the disease slowed significantly, with greater progression‐free survival in the arm receiving the highest supplementation. Moreover, in 2 years, CRC was 36% less likely to spread or be fatal in subjects who consumed more vitamin D.^63^ An updated meta‐analysis of randomized controlled trials confirmed that vitamin D supplementation significantly reduced cancer‐related mortality even though it did not reduce total cancer incidence.^63^

### 4.2 Effects through the gut microbiota and immune system

**FIGURE 2:**
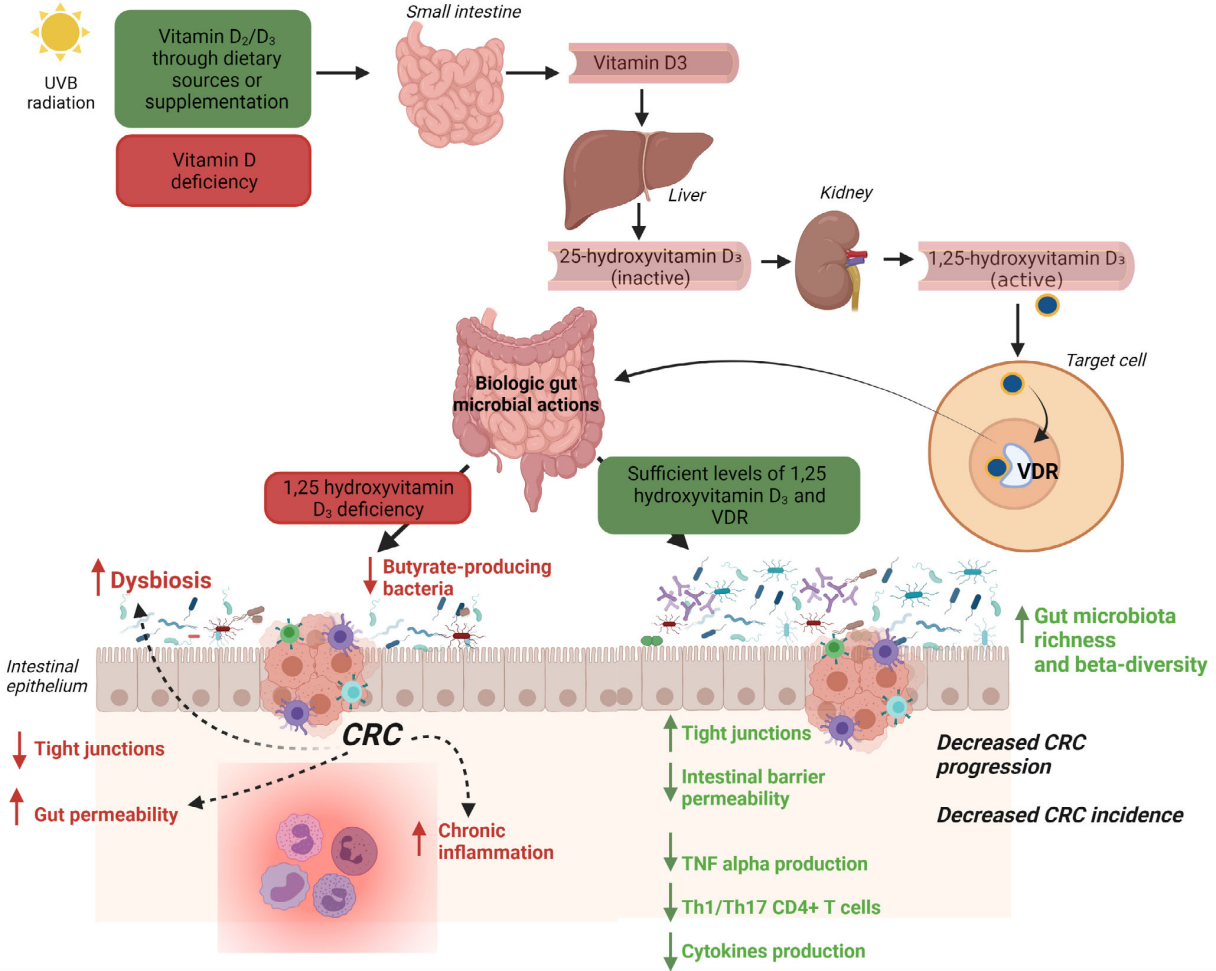
The impact of vitamin D intake in CRC process through gut microbiota modulation

Gut microbiota is a key driver of CRC development and progression: invasive pathobionts (e.g., *Escherichia coli*) may contribute to cancer onset counteracting autophagy and antimicrobial responses in epithelial colonic cells,^64^ and CRC patients have a pro‐inflammatory gut microbial signature, as stated above. Microbiota is, in turn, modulated by several nutrients such as the micronutrient vitamin D, through the immune system. Gut microbiota and immune system are strictly interconnected. The gut microbiota supports the development and response of the immune system, which in turn, regulates intestinal eubiosis balancing tolerance and immunity on the gut microbiota. Vitamin D plays a pivotal role in this complex network, since VDR is expressed (and activated under‐stimulation) in several immune cell lineages including CD4 and CD8 T cells, B cells, neutrophils, macrophages, and dendritic cells.^65^ In a human interventional study, vitamin D supplementation (a weekly dose of 980 IU/kg body weight of vitamin D3 for 8 weeks) significantly changed gut microbiome composition, reducing opportunistic pathogens and increasing bacterial richness. Specifically, the class of Gammaproteobacteria (including *Pseudomonas* spp and *Escherichia/Shigella* spp), significantly decreased. The effects on gut bacteria are supposed to be mediated by mucosal CD8+ T cells. These immune cells have a high expression of VDR: under vitamin D supplementation a switch from naïve to effector form occurs^66^; CD8+ T effector cells reduce the inflammatory environment through the calcitriol synthesis and allowing beneficial bacteria (such as Bacteroidetes) to outweigh opportunistic pathogens.^67^ These gut microbial modulations could counteract the gut barrier dysfunctions and dysbiosis seen during CRC development and progression. Conversely, vitamin D deficiency exacerbates gut microbiota dysfunctions triggered by CRC such as dysbiosis, decreases bacterial butyrate producers, and increases chronic inflammation leading to immunosuppression.^68^ A recent case–control study analyzed fecal microbiota composition of CRC and controls subjects evaluating the role of microbiome and diet (including vitamin D) in CRC etiology and regulation of inflammation markers.^69^ Researchers found an inverse association between CRC risk and high consumption of fatty fish (rich in omega‐3 polyunsaturated fatty acids and vitamin D). Conversely, a diet poor in fatty fish and rich in carbohydrates was significantly associated with CRC risk. The gut microbiome (*Bifidobacterium*/*Escherichia* genera ratio) was found to significantly mediate the effect of diet on CRC risk. Moreover, the gut microbial profile of CRC patients was enriched in pro‐inflammatory species such as *Parvimonas micra*, *F. nucleatum*, and *B. fragilis* species whereas controls specimens were associated with a higher abundance of *Bacteroidetes* and *Bifidobacterium* species. The link between gut inflammation and cancer has been well established.^70^ The risk of CRC increases with the duration of colitis in patients affected by inflammatory bowel diseases (IBD), correlating with the grade of inflammation and extend of the disease.^71^ Interestingly, a vitamin D deficit has been also related to IBDs onset, making this link ever closer.^72^ A human study correlated seasonal changes of circulating 25‐hydroxyvitamin D levels with the gut microbiome composition in IBD patients, observing a reduction in the abundance of bacterial genera typical for inflammation such as *Eggerthella lenta*, *Fusobacterium* spp, *Bacteroides* spp, *Collinsella aerofaciens*, and *Helicobacter* spp in summer/autumn period, when light exposure (and 1,25‐D synthesis) is higher.^73^ The immune cells are directly involved in the pathogenesis of both IBD and colitis‐associated CRC.^74^ In colon cancer cells, macrophage‐derived IL‐1β induces Wnt signaling.^75^ In this context, the 1,25‐D‐VDR binding inhibits the Wnt/β‐catenin activity in macrophages making them unable to activate Wnt signaling in colon cancer cells.^57^, ^63^ Moreover, chronic inflammation increases the turnover of epithelial cells and the release of ROS contributing to the development of low, high‐grade, and finally carcinoma lesions through the stepwise mutation of key genes in carcinogenesis such as DNA‐repair genes, p53, and KRAS.^76^ A recent mice model of inflammation‐associated colon cancer demonstrated that increased dietary vitamin D supplementation decreases inflammation, dysplasia, and tumor incidence, and is associated with decreased MAPK and NF‐κB expression during the acute inflammatory stage of disease.^77^ Cancer cells often contain abundant p53‐upregulated modulators of apoptosis (PUMA) interacting with VDR genes.^78^ VDR signaling is involved in the prevention of apoptosis by downregulating PUMA and blocking NF‐κB thus preventing the spread of damaged cells.^79^ In this context, the activity of VDR is also under the control of SCFAs, especially butyrate.^80^ This makes even closer the triple link between gut microbiota, Vitamin D, and the immune system in CRC. A mouse model study of colitis‐associated colon cancer^81^ went further, investigating the anti‐inflammatory, anti‐proliferative, and chemopreventive effects of vitamin D analog and assessing a down‐regulation of growth‐promoting c‐Myc gene, pro‐inflammatory COX‐2, and inhibition of ERK activation pathway in the premalignant phase. As regards CRC and the immune intestinal homeostasis, 1,25‐D promotes both antimicrobial activity and a tolerogenic response, as seen in experimental models of colitis.^50^ In detail, 1,25‐D inhibits in vitro and in vivo the interferon (IFN)‐γ and IL‐17 production from T cells,^82^ induces the anti‐inflammatory cytokine IL‐10 from Foxp3+ Treg cells^83^ and the antimicrobial IL‐22 from type 3 innate lymphoid (ILC3) cells.^84^ All these findings and statements allow us to attribute to vitamin D a preventive and anti‐proliferative role in CRC carcinogenesis (Figure 2), even if clinical studies with a prospective study design are still lacking in this setting.

## 5 CONCLUSIONS

In conclusion, vitamin D has been associated with CRC prevention and better clinical outcomes.^85^ Vitamin D acts as a regulatory prohormone with genetic and epigenetic targets, producing pleiotropic effects by the binding to its VDR, almost ubiquitous in the human body. In colonic mucosa, vitamin D may exert its action through an immune system modulation and microbial composition shaping, which, in turn, influences mucosal immunity. These effects influence the most known mechanisms of CRC development. To date, only preclinical and few clinical studies investigated the role of vitamin D as a chemopreventive agent in CRC. Further clinical studies with a large sample size and randomized design, are required to confirm the close interplay between vitamin D, microbiota, inflammation, and CRC, dissecting the role of vitamin D as a potential agent in CRC prevention and therapy.

## CONFLICT OF INTEREST

The authors declare no conflict of interest.

## Data Availability

Data sharing is not applicable to this article as no new data were created or analyzed in this study.

## References

[1] Feldman D , Krishnan AV , Swami S , Giovannucci E , Feldman BJ . The role of vitamin D in reducing cancer risk and progression. Nat Rev Cancer. 2014;14(5):342–57. 24705652 10.1038/nrc3691

[2] Holick MF . High prevalence of vitamin D inadequacy and implications for health. Mayo Clin Proc. 2006;81(3):353–73. 16529140 10.4065/81.3.353

[3] Welsh J , Wietzke JA , Zinser GM , Byrne B , Smith K , Narvaez CJ . Vitamin D‐3 receptor as a target for breast cancer prevention. J Nutr. 2003;133(7):2425S–33S. 12840219 10.1093/jn/133.7.2425S

[4] Herrmann M , Farrell C‐JL , Pusceddu I , Fabregat‐Cabello N , Cavalier E . Assessment of vitamin D status – a changing landscape. Clin Chem Lab Med. 2017;55(1):3–26. 27362963 10.1515/cclm-2016-0264

[5] Holick MF . Vitamin D deficiency. N Engl J Med. 2007;357(3):266–81. 17634462 10.1056/NEJMra070553

[6] Hossein‐nezhad A , Holick MF . Vitamin D for health: a global perspective. Mayo Clin Proc. 2013;88(7):720–55. 23790560 10.1016/j.mayocp.2013.05.011 PMC3761874

[7] Plum LA , DeLuca HF . Vitamin D, disease and therapeutic opportunities. Nat Rev Drug Discov. 2010;9(12):941–55. 21119732 10.1038/nrd3318

[8] Gandini S , Boniol M , Haukka J , Byrnes G , Cox B , Sneyd MJ , et al. Meta‐analysis of observational studies of serum 25‐hydroxyvitamin D levels and colorectal, breast and prostate cancer and colorectal adenoma. Int J Cancer. 2011;128(6):1414–24. 20473927 10.1002/ijc.25439

[9] Hossain S , Beydoun MA , Beydoun HA , Chen X , Zonderman AB , Wood RJ . Vitamin D and breast cancer: a systematic review and meta‐analysis of observational studies. Clin Nutr ESPEN. 2019;30:170–84. 30904218 10.1016/j.clnesp.2018.12.085 PMC6570818

[10] Theodoratou E , Tzoulaki I , Zgaga L , Ioannidis JPA . Vitamin D and multiple health outcomes: umbrella review of systematic reviews and meta‐analyses of observational studies and randomised trials. BMJ. 2014;348:g2035. 24690624 10.1136/bmj.g2035 PMC3972415

[11] Sun J . Dietary vitamin D, vitamin D receptor, and microbiome. Curr Opin Clin Nutr Metab Care. 2018;21(6):471–4. 30169457 10.1097/MCO.0000000000000516 PMC6168421

[12] Thursby E , Juge N . Introduction to the human gut microbiota. Biochem J. 2017;474(11):1823–36. 28512250 10.1042/BCJ20160510 PMC5433529

[13] Rinninella E , Raoul P , Cintoni M , Franceschi F , Miggiano GAD , Gasbarrini A , et al. What is the healthy gut microbiota composition? A changing ecosystem across age, environment, diet, and diseases. Microorganisms. 2019;7(1):14. 10.3390/microorganisms7010014 PMC6351938 30634578

[14] Liu Y , Baba Y , Ishimoto T , Iwatsuki M , Hiyoshi Y , Miyamoto Y , et al. Progress in characterizing the linkage between *Fusobacterium nucleatum* and gastrointestinal cancer. J Gastroenterol. 2019;54(1):33–41. 30244399 10.1007/s00535-018-1512-9

[15] Wang T , Cai G , Qiu Y , Fei N , Zhang M , Pang X , et al. Structural segregation of gut microbiota between colorectal cancer patients and healthy volunteers. ISME J. 2012;6(2):320–9. 21850056 10.1038/ismej.2011.109 PMC3260502

[16] Wirbel J , Pyl PT , Kartal E , Zych K , Kashani A , Milanese A , et al. Meta‐analysis of fecal metagenomes reveals global microbial signatures that are specific for colorectal cancer. Nat Med. 2019;25(4):679–89. 30936547 10.1038/s41591-019-0406-6 PMC7984229

[17] Qiao S , Lian X , Yue M , Zhang Q , Wei Z , Chen L , et al. Regulation of gut microbiota substantially contributes to the induction of intestinal Treg cells and consequent anti‐arthritis effect of madecassoside. Int Immunopharmacol. 2020;89(Pt A):107047. 33039960 10.1016/j.intimp.2020.107047

[18] Ng K , Nimeiri HS , McCleary NJ , Abrams TA , Yurgelun MB , Cleary JM , et al. Effect of high‐dose vs standard‐dose vitamin D3 supplementation on progression‐free survival among patients with advanced or metastatic colorectal cancer: the SUNSHINE randomized clinical trial. JAMA. 2019;321(14):1370–9. 30964527 10.1001/jama.2019.2402 PMC6459117

[19] Gasaly N , Hermoso MA , Gotteland M . Butyrate and the fine‐tuning of colonic homeostasis: implication for inflammatory bowel diseases. Int J Mol Sci. 2021;22(6):3061. 33802759 10.3390/ijms22063061 PMC8002420

[20] Usuda H , Okamoto T , Wada K . Leaky gut: effect of dietary fiber and fats on microbiome and intestinal barrier. Int J Mol Sci. 2021;22(14):7613. 34299233 10.3390/ijms22147613 PMC8305009

[21] Shen XJ , Rawls JF , Randall T , Burcal L , Mpande CN , Jenkins N , et al. Molecular characterization of mucosal adherent bacteria and associations with colorectal adenomas. Gut Microbes. 2010;1(3):138–47. 20740058 10.4161/gmic.1.3.12360 PMC2927011

[22] Kostic AD , Gevers D , Pedamallu CS , Michaud M , Duke F , Earl AM , et al. Genomic analysis identifies association of Fusobacterium with colorectal carcinoma. Genome Res. 2012;22(2):292–8. 22009990 10.1101/gr.126573.111 PMC3266036

[23] Tahara T , Yamamoto E , Suzuki H , Maruyama R , Chung W , Garriga J , et al. Fusobacterium in colonic flora and molecular features of colorectal carcinoma. Cancer Res. 2014;74(5):1311–8. 24385213 10.1158/0008-5472.CAN-13-1865 PMC4396185

[24] Lee D‐W , Han S‐W , Kang J‐K , Bae JM , Kim H‐P , Won J‐K , et al. Association between *Fusobacterium nucleatum*, pathway mutation, and patient prognosis in colorectal cancer. Ann Surg Oncol. 2018;25(11):3389–95. 30062471 10.1245/s10434-018-6681-5

[25] Rubinstein MR , Wang X , Liu W , Hao Y , Cai G , Han YW . *Fusobacterium nucleatum* promotes colorectal carcinogenesis by modulating E‐cadherin/β‐catenin signaling via its FadA adhesin. Cell Host Microbe. 2013;14(2):195–206. 23954158 10.1016/j.chom.2013.07.012 PMC3770529

[26] Tang B , Wang K , Jia Y‐P , Zhu P , Fang Y , Zhang Z‐J , et al. *Fusobacterium nucleatum*‐induced impairment of Autophagic flux enhances the expression of proinflammatory cytokines via ROS in Caco‐2 cells. PLoS One. 2016;11(11):e0165701. 27828984 10.1371/journal.pone.0165701 PMC5102440

[27] Abed J , Emgård JEM , Zamir G , Faroja M , Almogy G , Grenov A , et al. Fap2 mediates *Fusobacterium nucleatum* colorectal adenocarcinoma enrichment by binding to tumor‐expressed gal‐GalNAc. Cell Host Microbe. 2016;20(2):215–25. 27512904 10.1016/j.chom.2016.07.006 PMC5465824

[28] Yu X , Harden K , Gonzalez LC , Francesco M , Chiang E , Irving B , et al. The surface protein TIGIT suppresses T cell activation by promoting the generation of mature immunoregulatory dendritic cells. Nat Immunol. 2009;10(1):48–57. 19011627 10.1038/ni.1674

[29] Gur C , Ibrahim Y , Isaacson B , Yamin R , Abed J , Gamliel M , et al. Binding of the Fap2 protein of *Fusobacterium nucleatum* to human inhibitory receptor TIGIT protects tumors from immune cell attack. Immunity. 2015;42(2):344–55. 25680274 10.1016/j.immuni.2015.01.010 PMC4361732

[30] Kaplan CW , Ma X , Paranjpe A , Jewett A , Lux R , Kinder‐Haake S , et al. *Fusobacterium nucleatum* outer membrane proteins Fap2 and RadD induce cell death in human lymphocytes. Infect Immun. 2010;78(11):4773–8. 20823215 10.1128/IAI.00567-10 PMC2976331

[31] Wong SH , Zhao L , Zhang X , Nakatsu G , Han J , Xu W , et al. Gavage of fecal samples from patients with colorectal cancer promotes intestinal carcinogenesis in germ‐free and conventional mice. Gastroenterology. 2017;153(6):1621–1633.e6. 28823860 10.1053/j.gastro.2017.08.022

[32] Zackular JP , Baxter NT , Iverson KD , Sadler WD , Petrosino JF , Chen GY , et al. The gut microbiome modulates colon tumorigenesis. mBio. 2013;4(6):e00692–13. 24194538 10.1128/mBio.00692-13 PMC3892781

[33] Triner D , Devenport SN , Ramakrishnan SK , Ma X , Frieler RA , Greenson JK , et al. Neutrophils restrict tumor‐associated microbiota to reduce growth and invasion of colon tumors in mice. Gastroenterology. 2019;156(5):1467–82. 30550822 10.1053/j.gastro.2018.12.003 PMC6441634

[34] Kilkkinen A , Rissanen H , Klaukka T , Pukkala E , Heliövaara M , Huovinen P , et al. Antibiotic use predicts an increased risk of cancer. Int J Cancer. 2008;123(9):2152–5. 18704945 10.1002/ijc.23622

[35] Dik VK , van Oijen MGH , Smeets HM , Siersema PD . Frequent use of antibiotics is associated with colorectal cancer risk: results of a nested case–control study. Dig Dis Sci. 2016;61(1):255–64. 26289256 10.1007/s10620-015-3828-0 PMC4700063

[36] Willing BP , Russell SL , Finlay BB . Shifting the balance: antibiotic effects on host–microbiota mutualism. Nat Rev Microbiol. 2011;9(4):233–43. 21358670 10.1038/nrmicro2536

[37] Schett G , McInnes IB , Neurath MF . Reframing immune‐mediated inflammatory diseases through signature cytokine hubs. N Engl J Med. 2021;385(7):628–39. 34379924 10.1056/NEJMra1909094

[38] Gombart AF , Luong QT , Koeffler HP . Vitamin D compounds: activity against microbes and cancer. Anticancer Res. 2006;26(4A):2531–42. 16886661

[39] Kong J , Zhang Z , Musch MW , Ning G , Sun J , Hart J , et al. Novel role of the vitamin D receptor in maintaining the integrity of the intestinal mucosal barrier. Am J Physiol Gastrointest Liver Physiol. 2008;294(1):G208–16. 17962355 10.1152/ajpgi.00398.2007

[40] Zhang Y‐G , Wu S , Sun J . Vitamin D, Vitamin D receptor, and tissue barriers. Tissue Barriers. 2013;1(1):e23118. 24358453 10.4161/tisb.23118 PMC3865708

[41] Sun J , Kong J , Duan Y , Szeto FL , Liao A , Madara JL , et al. Increased NF‐κB activity in fibroblasts lacking the vitamin D receptor. Am J Physiol Endocrinol Metabol. 2006;291(2):E315–22. 10.1152/ajpendo.00590.2005 16507601

[42] Wu S , Xia Y , Liu X , Sun J . Vitamin D receptor deletion leads to reduced level of IkappaBalpha protein through protein translation, protein‐protein interaction, and post‐translational modification. Int J Biochem Cell Biol. 2010;42(2):329–36. 19931640 10.1016/j.biocel.2009.11.012 PMC2818560

[43] Adams JS , Hewison M . Unexpected actions of vitamin D: new perspectives on the regulation of innate and adaptive immunity. Nat Clin Pract Endocrinol Metab. 2008;4(2):80–90. 18212810 10.1038/ncpendmet0716 PMC2678245

[44] Merriman KE , Kweh MF , Powell JL , Lippolis JD , Nelson CD . Multiple β‐defensin genes are upregulated by the vitamin D pathway in cattle. J Steroid Biochem Mol Biol. 2015;154:120–9. 26255277 10.1016/j.jsbmb.2015.08.002

[45] Raftery T , Martineau AR , Greiller CL , Ghosh S , McNamara D , Bennett K , et al. Effects of vitamin D supplementation on intestinal permeability, cathelicidin and disease markers in Crohn's disease: results from a randomised double‐blind placebo‐controlled study. United Eur Gastroenterol J. 2015;3(3):294–302. 10.1177/2050640615572176 PMC4480538 26137304

[46] Guo C , Sinnott B , Niu B , Lowry MB , Fantacone ML , Gombart AF . Synergistic induction of human cathelicidin antimicrobial peptide gene expression by vitamin D and stilbenoids. Mol Nutr Food Res. 2014;58(3):528–36. 24039193 10.1002/mnfr.201300266 PMC3947465

[47] Diamond G , Beckloff N , Weinberg A , Kisich KO . The roles of antimicrobial peptides in innate host defense. Curr Pharm Des. 2009;15(21):2377–92. 19601838 10.2174/138161209788682325 PMC2750833

[48] Kamen DL , Tangpricha V . Vitamin D and molecular actions on the immune system: modulation of innate and autoimmunity. J Mol Med. 2010;88(5):441–50. 10.1007/s00109-010-0590-9 20119827 PMC2861286

[49] Chatterjee I , Lu R , Zhang Y , Zhang J , Dai Y , Xia Y , et al. Vitamin D receptor promotes healthy microbial metabolites and microbiome. Sci Rep. 2020;10(1):7340. 32355205 10.1038/s41598-020-64226-7 PMC7192915

[50] Cantorna MT , Snyder L , Arora J . Vitamin A and vitamin D regulate the microbial complexity, barrier function, and the mucosal immune responses to ensure intestinal homeostasis. Crit Rev Biochem Mol Biol. 2019;54(2):184–92. 31084433 10.1080/10409238.2019.1611734 PMC6629036

[51] Wang J , Thingholm LB , Skiecevičienė J , Rausch P , Kummen M , Hov JR , et al. Genome‐wide association analysis identifies variation in vitamin D receptor and other host factors influencing the gut microbiota. Nat Genet. 2016;48(11):1396–406. 27723756 10.1038/ng.3695 PMC5626933

[52] Vitamin D Signaling through Induction of Paneth Cell Defensins Maintains Gut Microbiota and Improves Metabolic Disorders and Hepatic Steatosis in Animal Models—PubMed [Internet]. [cited 2021 Jun 15]. Available from: https://pubmed.ncbi.nlm.nih.gov/27895587/ 10.3389/fphys.2016.00498 PMC5108805 27895587

[53] Naderpoor N , Mousa A , Fernanda Gomez Arango L , Barrett HL , Dekker Nitert M , de Courten B . Effect of vitamin D supplementation on faecal microbiota: a randomised clinical trial. Nutrients. 2019;11(12):2888. 10.3390/nu11122888 PMC6950585 31783602

[54] Singh P , Rawat A , Alwakeel M , Sharif E , Al KS . The potential role of vitamin D supplementation as a gut microbiota modifier in healthy individuals. Sci Rep. 2020;10(1):21641. 33303854 10.1038/s41598-020-77806-4 PMC7729960

[55] Thomas RL , Jiang L , Adams JS , Xu ZZ , Shen J , Janssen S , et al. Vitamin D metabolites and the gut microbiome in older men. Nat Commun. 2020;11(1):5997. 33244003 10.1038/s41467-020-19793-8 PMC7693238

[56] Waterhouse M , Hope B , Krause L , Morrison M , Protani MM , Zakrzewski M , et al. Vitamin D and the gut microbiome: a systematic review of in vivo studies. Eur J Nutr. 2019;58(7):2895–910. 30324342 10.1007/s00394-018-1842-7

[57] Fedirko V , Bostick RM , Long Q , Flanders WD , McCullough ML , Sidelnikov E , et al. Effects of supplemental vitamin D and calcium on oxidative DNA damage marker in normal colorectal mucosa: a randomized clinical trial. Cancer Epidemiol Biomarkers Prev. 2010;19(1):280–91. 20056649 10.1158/1055-9965.EPI-09-0448 PMC2805163

[58] Carlberg C , Muñoz A . An update on vitamin D signaling and cancer. Semin Cancer Biol. 2020. Online ahead of print. 10.1016/j.semcancer.2020.05.018 32485310

[59] Sheinin Y , Kaserer K , Wrba F , Wenzl E , Kriwanek S , Peterlik M , et al. In situ mRNA hybridization analysis and immunolocalization of the vitamin D receptor in normal and carcinomatous human colonic mucosa: relation to epidermal growth factor receptor expression. Virchows Arch. 2000;437(5):501–7. 11147170 10.1007/s004280000275

[60] Alvarez‐Díaz S , Valle N , García JM , Peña C , Freije JMP , Quesada V , et al. Cystatin D is a candidate tumor suppressor gene induced by vitamin D in human colon cancer cells. J Clin Invest. 2009;119(8):2343–58. 19662683 10.1172/JCI37205 PMC2719930

[61] Alvarez‐Díaz S , Valle N , Ferrer‐Mayorga G , Lombardía L , Herrera M , Domínguez O , et al. MicroRNA‐22 is induced by vitamin D and contributes to its antiproliferative, antimigratory and gene regulatory effects in colon cancer cells. Hum Mol Genet. 2012;21(10):2157–65. 22328083 10.1093/hmg/dds031

[62] Protiva P , Cross HS , Hopkins ME , Kállay E , Bises G , Dreyhaupt E , et al. Chemoprevention of colorectal neoplasia by estrogen: potential role of vitamin D activity. Cancer Prev Res. 2009;2(1):43–51. 10.1158/1940-6207.CAPR-08-0103 19139017

[63] Keum N , Lee DH , Greenwood DC , Manson JE , Giovannucci E . Vitamin D supplementation and total cancer incidence and mortality: a meta‐analysis of randomized controlled trials. Ann Oncol. 2019;30(5):733–43. 30796437 10.1093/annonc/mdz059 PMC6821324

[64] Chia‐Hui Yu L , Wei S‐C , Li Y‐H , Lin P‐Y , Chang X‐Y , Weng J‐P , et al. Invasive pathobionts contribute to colon cancer initiation by counterbalancing epithelial antimicrobial responses. Cell Mol Gastroenterol Hepatol. 2021. Online ahead of print. 10.1016/j.jcmgh.2021.08.007 PMC8600093 34418587

[65] Malaguarnera L . Vitamin D and microbiota: two sides of the same coin in the immunomodulatory aspects. Int Immunopharmacol. 2020;79:106112. 31877495 10.1016/j.intimp.2019.106112

[66] Hwang YG , Hsu H‐C , Lim F‐C , Wu Q , Yang P , Fisher G , et al. Increased vitamin D is associated with decline of naïve, but accumulation of effector, CD8 T cells during early aging. Adv Aging Res. 2013;2(2):72–80. 25392765 10.4236/aar.2013.22010 PMC4226219

[67] Bashir M , Prietl B , Tauschmann M , Mautner SI , Kump PK , Treiber G , et al. Effects of high doses of vitamin D3 on mucosa‐associated gut microbiome vary between regions of the human gastrointestinal tract. Eur J Nutr. 2016;55(4):1479–89. 26130323 10.1007/s00394-015-0966-2 PMC4875045

[68] Tangestani H , Boroujeni HK , Djafarian K , Emamat H , Shab‐Bidar S . Vitamin D and the gut microbiota: a narrative literature review. Clin Nutr Res. 2021;10(3):181–91. 34386438 10.7762/cnr.2021.10.3.181 PMC8331286

[69] Serrano D , Pozzi C , Guglietta S , Fosso B , Suppa M , Gnagnarella P , et al. Microbiome as mediator of diet on colorectal cancer risk: the role of vitamin D. Markers of inflammation and adipokines. Nutrients. 2021;13(2):363. 10.3390/nu13020363 33504116 PMC7911673

[70] Dulai PS , Sandborn WJ , Gupta S . Colorectal cancer and dysplasia in inflammatory bowel disease: a review of disease epidemiology, pathophysiology, and management. Cancer Prev Res (Phila). 2016;9(12):887–94. 27679553 10.1158/1940-6207.CAPR-16-0124 PMC5289746

[71] Grivennikov SI . Inflammation and colorectal cancer: colitis‐associated neoplasia. Semin Immunopathol. 2013;35(2):229–44. 23161445 10.1007/s00281-012-0352-6 PMC3568220

[72] Raftery T , Merrick M , Healy M , Mahmud N , O'Morain C , Smith S , et al. Vitamin D status is associated with intestinal inflammation as measured by fecal calprotectin in Crohn's disease in clinical remission. Dig Dis Sci. 2015;60(8):2427–35. 25757449 10.1007/s10620-015-3620-1

[73] Soltys K , Stuchlikova M , Hlavaty T , Gaalova B , Budis J , Gazdarica J , et al. Seasonal changes of circulating 25‐hydroxyvitamin D correlate with the lower gut microbiome composition in inflammatory bowel disease patients. Sci Rep. 2020;10(1):6024. 32265456 10.1038/s41598-020-62811-4 PMC7138827

[74] Sakai K , De Velasco MA , Kura Y , Nishio K . Transcriptome profiling and metagenomic analysis help to elucidate interactions in an inflammation‐associated cancer mouse model. Cancers (Basel). 2021;13(15):3683. 34359585 10.3390/cancers13153683 PMC8345192

[75] Kaler P , Augenlicht L , Klampfer L . Macrophage‐derived IL‐1b stimulates Wnt signaling and growth of colon cancer cells: a crosstalk interrupted by vitamin D3. Oncogene. 2009;28(44):3892–902. 19701245 10.1038/onc.2009.247 PMC2783659

[76] Rogler G . Chronic ulcerative colitis and colorectal cancer. Cancer Lett. 2014;345(2):235–41. 23941831 10.1016/j.canlet.2013.07.032

[77] Meeker S , Seamons A , Paik J , Treuting PM , Brabb T , Grady WM , et al. Increased dietary vitamin D suppresses MAPK signaling, colitis, and colon cancer. Cancer Res. 2014;74(16):4398–408. 24938764 10.1158/0008-5472.CAN-13-2820 PMC4134774

[78] Stambolsky P , Tabach Y , Fontemaggi G , Weisz L , Maor‐Aloni R , Siegfried Z , et al. Modulation of the vitamin D3 response by cancer‐associated mutant p53. Cancer Cell. 2010;17(3):273–85. 20227041 10.1016/j.ccr.2009.11.025 PMC2882298

[79] He L , Liu T , Shi Y , Tian F , Hu H , Deb DK , et al. Gut epithelial vitamin D receptor regulates microbiota‐dependent mucosal inflammation by suppressing intestinal epithelial cell apoptosis. Endocrinology. 2018;159(2):967–79. 29228157 10.1210/en.2017-00748 PMC5788002

[80] Schwab M , Reynders V , Loitsch S , Steinhilber D , Stein J , Schröder O . Involvement of different nuclear hormone receptors in butyrate‐mediated inhibition of inducible NF kappa B signalling. Mol Immunol. 2007;44(15):3625–32. 17521736 10.1016/j.molimm.2007.04.010

[81] Fichera A , Little N , Dougherty U , Mustafi R , Cerda S , Li YC , et al. A vitamin D analogue inhibits colonic carcinogenesis in the AOM/DSS model. J Surg Res. 2007;142(2):239–45. 17574271 10.1016/j.jss.2007.02.038

[82] Lemire JM . Immunomodulatory role of 1,25‐dihydroxyvitamin D3. J Cell Biochem. 1992;49(1):26–31. 1644850 10.1002/jcb.240490106

[83] Kang SW , Kim SH , Lee N , Lee W‐W , Hwang K‐A , Shin MS , et al. 1,25‐Dihyroxyvitamin D3 promotes FOXP3 expression via binding to vitamin D response elements in its conserved noncoding sequence region. J Immunol. 2012;188(11):5276–82. 22529297 10.4049/jimmunol.1101211 PMC3358577

[84] Lin Y‐D , Arora J , Diehl K , Bora SA , Cantorna MT . Vitamin D is required for ILC3 derived IL‐22 and protection from *Citrobacter rodentium* infection. Front Immunol. 2019;10:1. 30723466 10.3389/fimmu.2019.00001 PMC6349822

[85] Boughanem H , Canudas S , Hernandez‐Alonso P , Becerra‐Tomás N , Babio N , Salas‐Salvadó J , et al. Vitamin D intake and the risk of colorectal cancer: an updated meta‐analysis and systematic review of case–control and prospective cohort studies. Cancers (Basel). 2021;13(11):2814. 34200111 10.3390/cancers13112814 PMC8201292

